# Progressive Nephron Loss in Aging Kidneys: Clinical–Structural Associations Investigated by Two Anatomical Methods

**DOI:** 10.1002/ar.24249

**Published:** 2019-10-09

**Authors:** Michael D. Hughson, Wendy E. Hoy, John F. Bertram

**Affiliations:** ^1^ University of Mississippi Medical Center Jackson Mississippi; ^2^ Shorsh General Hospital Sulaimaniyah Iraq; ^3^ Centre for Chronic Disease University of Queensland Brisbane Queensland Australia; ^4^ Cardiovascular Disease Program, Biomedicine Discovery Institute and Department of Anatomy and Developmental Biology Monash University Clayton Victoria Australia

**Keywords:** aging, nephrosclerosis, glomerulosclerosis, stereology, birth weight

## Abstract

Two major studies of structural changes associated with aging in human kidneys are reviewed and new information presented. The studies are the Monash University stereologically analyzed series of 319 autopsy kidneys from the United States in which 44% were white and the Mayo Clinic CT angiogram/biopsy analysis of 1,388 US kidney donors in which 97% were white. Hypertension rates in the Monash series were 48% and included moderate and severe hypertension. In the Mayo Clinic study, 12% had mild hypertension. The studies showed no relationship between glomerular number and hypertension except for a weak relationship with older white women in the Monash series. An inverse relationship was present between glomerular number and glomerular volume, a reciprocity that tended to enhance glomerular mass and by inference filtration capacity with lower nephron numbers. This relationship seemed to be present whether low nephron numbers were intrinsic or acquired. In the Mayo Clinic studies, pretransplant iothalamate clearances demonstrated that single nephron (SN) glomerular filtration rates (GFR) were similar throughout the range of glomerular number in subjects younger than 70 years, but that increased SNGFR correlated with nephron hypertrophy and increased nephrosclerosis particularly at 70 years of age and over. Hypertension at least through middle age cannot be related to a deficiency of glomeruli, but glomeruli are lost with later aging in association with adaptive nephron hypertrophy that can maintain GFR near normal. These studies help define an age‐related nephropathy that overlaps with hypertension as a potential cause of end‐stage renal disease when glomerulosclerosis is advanced.

## 1 INTRODUCTION

Brenner et al. (1988), on the basis of clinical and epidemiologic observations, suggested that essential hypertension may be related to a nephron deficit secondary to low birth weight or to glomerular loss during adulthood. The concept was partly derived from the studies of Barker et al. (1989) who found that low birth weight segregated into communities with high rates of stroke and ischemic heart disease and also predicted hypertension that began in young adults and increased with age. The adverse effects of low birth weight on aging adults includes obesity, hypertension, cardiovascular disease (CVD), and Type 2 diabetes that in various combinations have a major influence on the kidney (Eckel et al., 2005). If susceptible persons can avoid early ischemic heart disease death, they are at risk of developing chronic kidney disease that can be diabetic, hypertensive, obesity‐related, or a chronic scarring arteriosclerotic nephropathy without hypertension.

In the early 1990s, Hoy et al. (1998, 1999) began to investigate kidney disease of Tiwi Islanders, an Australian Aboriginal group at very high risk of Type 2 diabetes and end‐stage renal disease (ESRD) who also had very high rates of low birth weight. The kidney disease was found to be multifactorial (Hoy et al., 2012). It was partly the result of infection directly or remotely involving the kidney but partly associated with low birth weight, diabetes, and obesity with the contributing factors being attributed to the disadvantaged circumstances of the communities (Hoy et al., 2012). The findings tended to confirm the “Barker hypothesis” that intrauterine factors influenced the development of adult disease, and in stereological studies performed by Bertram at Monash University, Aboriginal Australians were found to have much lower numbers of glomeruli than Australians of European descent (Hoy et al., 2006).

The early studies emphasized Aboriginal renal disease but then developed into an international project that explored the relationship between nephron number, blood pressure, and birth weight among multiracial Mississippi residents that have among the highest rates of diabetes, obesity, and hypertension in the United States (Young et al., 2000; Hoy et al., 2003, Hughson et al., 2003; Hughson et al., 2006). Later, relationships between nephron number, glomerular size, hypertension, and single nephron glomerular filtration rates (SNGFR) were explored among the Japanese who, with the Taiwanese, have the longest life‐expectancy and the highest rates of ESRD in the world (Kanzaki et al., 2017). These studies comprised a more than 20‐year effort that investigated ethnic differences in kidney anatomy among US whites and African Americans, Australian whites and Aborigines, Senegalese Africans (McNamara et al., 2010), and the Japanese.

In this review, the application of the disector/ fractionator stereological method used in the Monash series will be examined together with computerized tomography (CT) in conjunction with renal biopsies (CT/biopsy) used by the Mayo Clinic group. The disector/fractionator is used primarily at autopsy, whereas the CT/biopsy method can be conducted on living persons. These studies have clinical significance because low nephron endowment may help define patients at high risk for progressive chronic kidney disease who may benefit from early clinical intervention to prevent ESRD (Whaley‐Connell, et al., 2008). The review will be primarily about the US cases because many more kidneys have been analyzed from the US than from elsewhere. The Monash series have been updated to include subjects that were not included in previous publications. The Japanese and Australian cohorts have been the subject of recent publications and will not be considered here (Hoy et al., 2016; Hoy et al., 2017; Kanzaki et al., 2017). Neither will the effects of prematurity and very low birth weight be covered. This recently has been intensively covered as a factor in Aboriginal renal disease by Hoy et al. (2017).

## 2 METHODS

This research used human tissue. The study was approved by the Institutional Review Board of the University of Mississippi Medical Center and the Human Research Ethics Committee of Monash University. Permission for autopsy was obtained from the next of kin.

The earliest determinations of glomerular number used acid maceration to separate nephron segments from a known volume of cortex and counted the glomeruli with the final estimate extrapolating the volume of the macerated tissue to the total volume of renal cortex (Dunnill and Halley, 1973; McLachlan et al., 1977). This produced glomerular numbers that were not notably different from newer techniques used today, but the numbers of cases studied were limited as reviewed by Denic et al. (2017a).

### 2.1 The Disector/Fractionator Combination

The gold standard for counting glomeruli is currently the disector/fractionator stereological method (Bertram, 1995; Cullen‐McEwen et al., 2012). “Disector” refers to using pairs of histological sections (two sections at a time) to sample (count) glomeruli, while “fractionator” refers to the fact that glomeruli are counted in a known fraction of cortical tissue.

The disector/fractionator is a design‐based stereological method that depends upon a careful, complete slicing of a fixed kidney, and then obtaining a known fraction of cortical tissue through systematic, uniform random sampling. The first fraction is obtained by slicing a kidney of known weight into half and then one half into 4 mm slices. One in four of these 4 mm slices are sampled and weighed. The weight of these sampled slices as a fraction of the weight of the whole organ is the first “weight” fraction. The medulla is then removed, and each sampled slice is cut to produce sample blocks that are generally squares about 0.1 cm thick and 1 cm on each side. A known fraction of these sample blocks is then selected (usually 1 in 20) yielding the second “slice” sampling fraction. The location of the samples from the cortex can be determined by the subsequent histologic sections showing capsule, a rim of upper medulla, or none.

**Figure 1:**
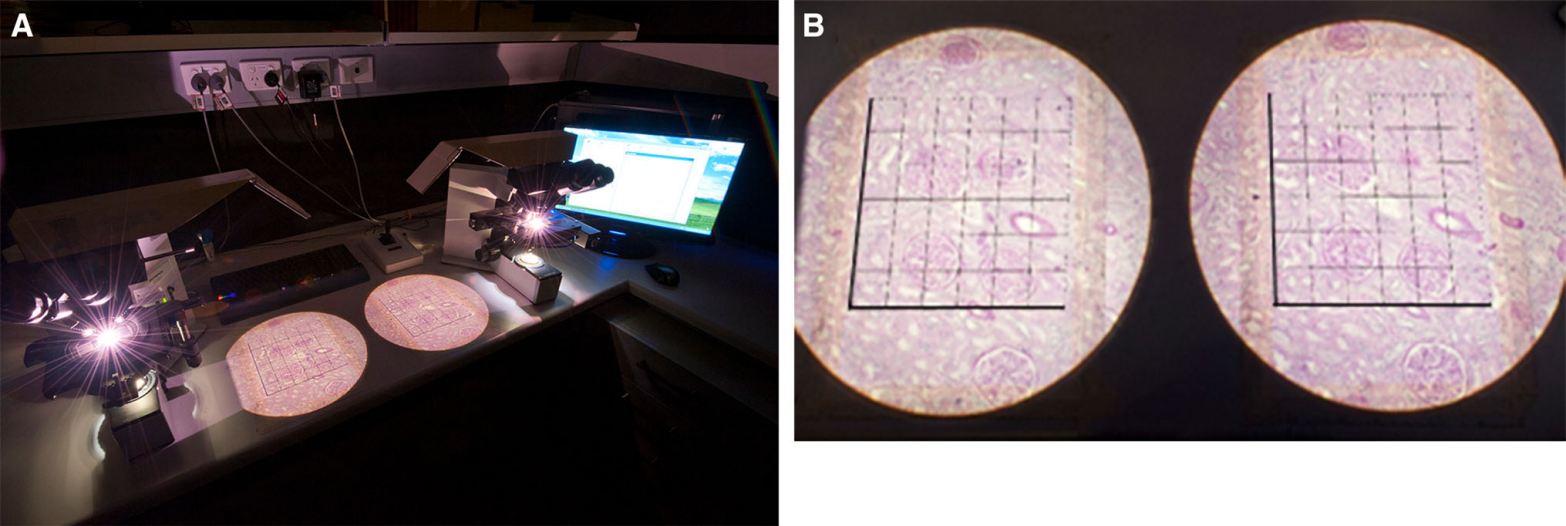
(**A**). The physical disector apparatus used to estimate glomerular number in the Monash series. This consists of tandem microscopes projecting images of a pair of histologic sections from tissue blocks representing a known fraction of the kidney cortex. (**B**). Identical areas of the paired sections are projected side by side. On the right is the “reference” section with an unbiased counting frame that analyzes glomeruli not touching the forbidden (dark) lines. The “look‐up” section is on the left. A glomerulus present in the unbiased counting frame but not in the look‐up section is counted

The selected sample blocks are embedded in glycol methacrylate to avoid the shrinkage of processing into paraffin and then completely serially sectioned at a thickness of 20 μm to produce approximately 100–150 section pairs per kidney. Every 10th and 11th paired sections are selected producing a “section” sampling fraction. The 10th (“reference” section) is examined with the 11th “look‐up” section. Identical regions of the section pairs are projected onto unbiased counting frames (grids) from tandem microscopes (Fig. 1A), so that a glomerulus that is seen on the reference section can be identified as having disappeared in the look‐up section (Fig. 1B).

The number of disappearing glomeruli in the serial sections represents the number of glomeruli in the block. From the individual blocks representing a known fraction of the whole cortex, the total number of glomeruli (N_glom_) in the kidney is calculated. The glomerular tuft areas are calculated from the points that they overly on the grid. The average glomerular volume (V_glom_) is derived from the volume density divided by the numerical density of glomeruli in the kidney. Multiplication of N_glom_ by V_glom_ provides an estimate of the total glomerular volume of the kidney (V_glom_total).

Counting disappearing glomeruli is tricky and requires someone with keen observational skills, but the procedure is conceptually straightforward, and the results are considered design‐based because they are not influenced by variations in glomerular tuft shape and importantly by differences in glomerular size that can vary considerably within a single kidney (Hoy et al., 2010). For this reason, counts are often referred to as “unbiased,” but this is only the case if the technique is used correctly.

The percent of glomerulosclerosis, the severity of arteriosclerosis, and the percent of interstitial fibrosis and tubular atrophy were analyzed morphometrically as planar data with Image ProPlus software as described previously (Hughson et al., 2014; Hughson et al., 2016). The analysis of arteriosclerosis measured arterial intimal thickening in large intrarenal arteries at the arcuate and distal interlobar level (>250 μm in diameter) and in the small interlobular arteries (90–250 μm in diameter). Because few of the continuous variables were normally distributed, data are presented as median and interquartile ranges (IQRs), and two‐way differences are analyzed by Wilcoxon rank sum tests.

### 2.2 Renal CT with Angiogram Enhancement and Biopsy

**Figure 2:**
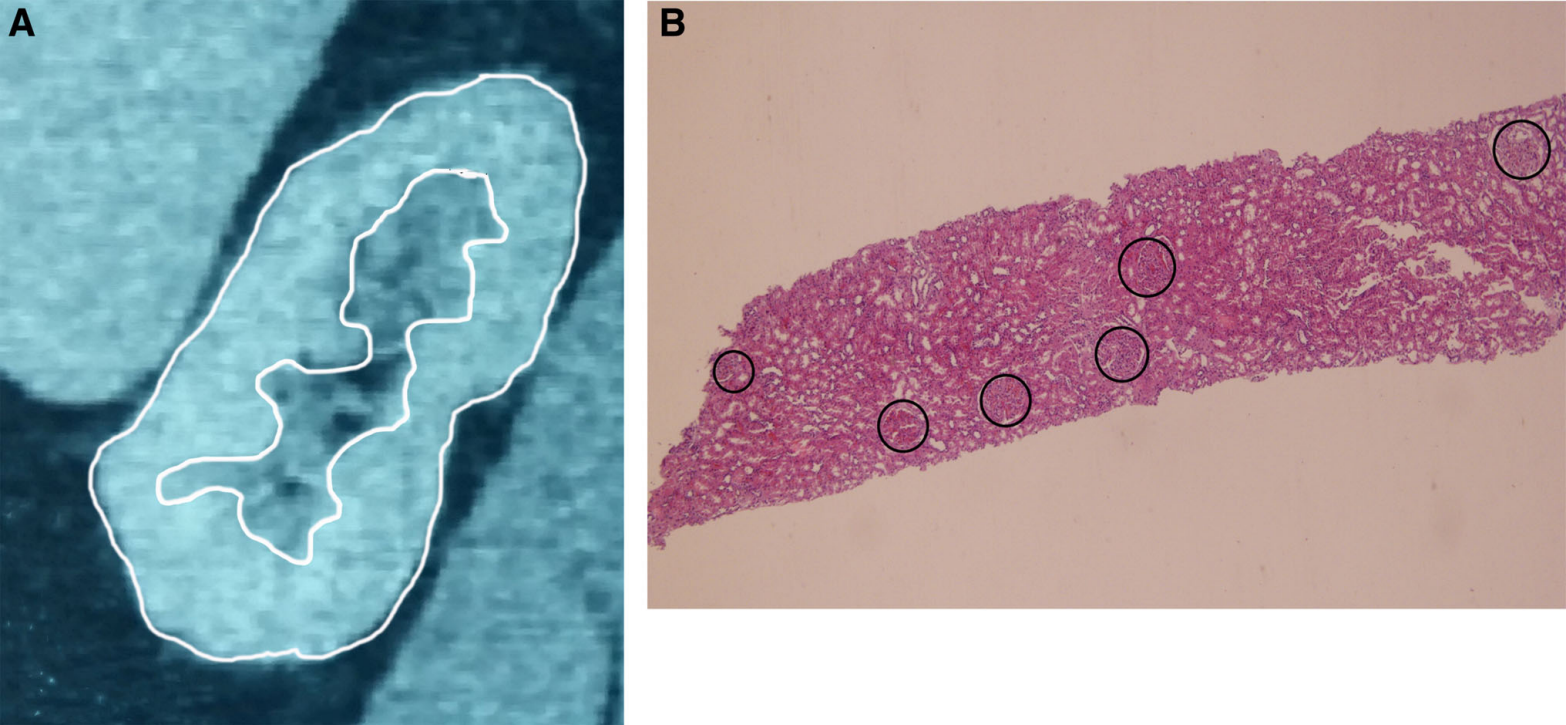
Enhanced CT and renal biopsy. (**A**). CT of a kidney with the cortex outlined in white. Serial reconstruction then allows an estimate of total cortical volume. (**B**). A renal biopsy 8.0 × 1.8 mm contains six glomeruli for a glomerular density of 0.42/mm^2^. Mean glomerular area for the biopsy is measured, and volume and density are estimated in three dimensions by Weibel and Gomez formulae (1962)

The disector/fractionator method is a prolonged procedure that at best can produce results for 2–3 human kidneys a week. In humans, it is only applicable to autopsy or surgically removed kidneys that are not to be transplanted. Recently, the Mayo Clinic group has developed a biopsy sampling method that has been used to study a large number of kidney donors (Denic et al., 2017a, 2017b). The method involves a calculation of cortical volume using contrast‐enhanced CT and then a renal biopsy to determine glomerular density and glomerular and tubular area by digital imaging on single histologic sections (Fig. 2A, B).

These determinations are calculated as:

$$\text{Area glomerular density}\;\left(\text{number of glomeruli}/{\mathrm{mm}}^{2}\right)=\text{number of glomeruli in the biopsy section divided}\;\mathrm{by}\;\text{the area of cortex in the biopsy}.$$

$$\text{Mean glomerular area}\;\left({\mathrm{mm}}^{2}\right)=\text{total area of the glomerular cross sections in the biopsy divided}\;\mathrm{by}\;\text{the number of glomeruli in the section}.$$

Mean glomerular volume and glomerular number are then calculated per mm^3^ of tissue using the formulae of Weibel and Gomez (1962):

$$\text{Mean glomerular volume}\;\left({\mathrm{mm}}^{3}\right)=\left[1.382\times {\left(\text{mean glomerular area}\right)}^{3/2}/1.01\right].$$

$$\text{Glomerular density}\;\mathrm{per}\;{\mathrm{mm}}^{3}\;\text{cortex}=1/1.382\times 2\surd {\left(\text{area glomerular density}\right)}^{3}/\text{mean glomerular area}.$$

$$\text{The total number of glomeruli in the kidney}=\left[\text{glomerular density of cortex}/{\mathrm{mm}}^{3}\times \text{total cortical volume}\;\mathrm{as}\;\text{determined}\;\mathrm{by}\;\text{enhanced}\;\mathrm{CT}\right]\;\text{divided}\;\mathrm{by}\;1.268\times 1.43.$$

The calculations use four correction factors: 1.382 is a correction factor that assumes all glomeruli are spheres; 1.01 is a correction factor for glomerular size heterogeneity. The factor of 1.268 corrects for volume shrinkage due to loss of perfusion pressure, and 1.43 corrects for tissue volume shrinkage resulting from formalin fixation and paraffin embedding (Lerman et al., 1990; Fulladosa et al., 2003).

Iothalamate GFRs were measured at Mayo Clinic as a standard of care in the pre‐donation evaluation of living kidney donors, and pretransplant SNGFRs were estimated.

## 3 RESULTS

### 3.1 Disector/Fractionator Stereological Analysis

The Monash series, consisted of right kidneys from 180 African Americans and 139 whites having an age range from 5 weeks to 89 years old. The median age was 41.0 years with an IQR (25th to 75th percentile) of 29–51 years. To prevent excessive glomerular loss due to renal scarring from interfering with glomerular counting, the collection excluded diseased kidneys or kidneys with any more than moderate arteriolonephrosclerosis. This restriction resulted in the collection of only 20 subjects 65 years or older. Clinical information on the subjects included cause of death, hypertension history, and height and weight from which body mass index (BMI as kg/m^2^) was calculated. The causes of death among this autopsy cohort closely resemble that of the general Mississippi population with approximately one‐third of deaths being caused by CVD (Hughson et al., 2017).

Birth weights were obtained on 202 subjects from the Mississippi State Health Department. The Health Department began recording birth weights in 1953, making the age range limited to subjects 55 years or older by the time the collection was completed in 2008. In Table 1, these clinical characteristics are tabulated by race together with the total glomerular number of the right kidney and the average glomerular volume, designated respectively N_glom_ and V_glom_ for the Monash series.

**Table 1:** Characteristic of US autopsy with stereological and morphometric findings

Characteristic	African American	White	*P*‐value
Subjects, all (n)	180	139	
Age ≥18 years (n)	152	131	0.01
Age, all (years)	38.5 (25–48)	43 (32–53)	0.001
Age ≥18 years	42 (34–51)	44 (36–54)	0.05
Male	0.48	0.63	0.01
BMI age ≥ 18 years	28.2 (25–34)	27 (24–34)	0.48
BMI ≥30 (≥18 years)	0.45	0.42	0.85
CVD death (≥18 years)	0.37	0.35	0.75
Hypertension (≥18 years)	0.56	0.37	0.001
Birth weight (kg)	3.25 (2.80–3.72)	3.25 (2.85–3.46)	0.67
N_glom_	868,983 (92,639–1,050,758)	905,102 (712,674–1,135,031)	0.29
V_glom_ (≥18 years)	7.59 (5.79–9.55)	6.58 (4.93–8.22)	<0.001
Glomerulosclerosis (%)	1.9 (0.8–5.4)	1.7 (0.8–3.3)	0.35
Cortical fibrosis (%)	3.9 (1.6–8.1)	3.1 (1.4–6.1)	0.10
Arteriosclerosis (%), small	6.9 (2.2–14.4)	4.3 (1.3–8.8)	0.002
Arteriosclerosis (%), large	11.7 (6.0–18.4)	11.5 (6.1–15.2)	0.06

Continuous variable are expressed as median and (IQR) and discrete variables as frequency.

BMI, body mass index (kg/m^2^); N_glom_, number of glomeruli in right kidney; V_glom_, mean glomerular volume (μm^3^ × 10^6^); Arteriosclerosis (%), small, percent of arterial intimal thickening in small (interlobular) renal arteries; Arteriosclerosis (%), large, percent of arterial intimal thickening in large (arcuate) renal arteries.

White subjects were older than African Americans overall and for those 18 years or older. This reflected the greater numbers of neonatal and childhood deaths among African Americans as well as an earlier age of CVD deaths among African Americans (Hoy et al., 2015; Hughson et al., 2017). The earlier CVD deaths were found in persons having one or two apolipoprotein L1 (APOL1) risk‐alleles (Hughson et al., 2017). APOL1 risk‐alleles are gene variants associated with a high frequency of ESRD related to hypertension or focal segmental glomerulosclerosis among persons of African descent, and it was notable that the gene variants were also found in African Americans with premature adult CVD deaths unrelated to hypertension (Hughson et al., 2017).

The median N_glom_ for all subjects was 884,609 (IQR, 708,976–1,110,410) with a more than 9‐fold range from 210,332 to 2,702,079. There was no significant racial difference (*P* = 0.29), but females had significantly fewer glomeruli than males [males 932,850 (IQR, 743,442–1,169,966); females 827,693 (IQR, 677,531–994,279), *P* = 0.001].

**Figure 3:**
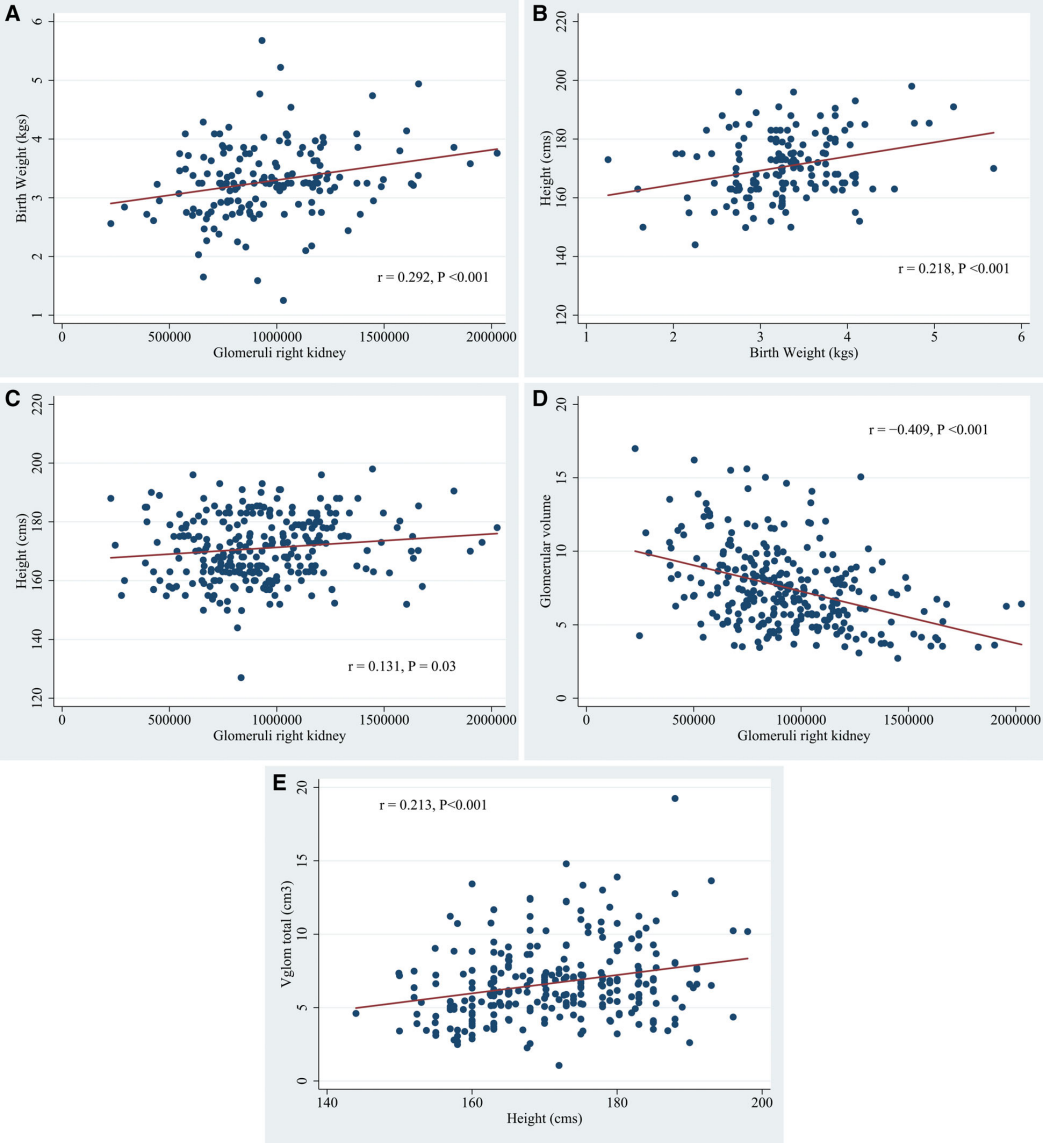
From the Monash series study of the US adults (≥18 years old). Significant direct relationships were found between birth weight and glomerular number (**A**), birth weight and adult height (**B**), and adult height and glomerular number (**C**). There is a strong inverse relationship between total glomerular number and mean glomerular volume in which lower numbers of glomeruli predict larger glomerular size (**D**). The product of total glomerular number and mean glomerular volume represents total glomerular volume (V_glom_total), an estimate of the filtration capacity of the kidney. V_glom_total increases with height (**E**) in a manner that somewhat attenuates the glomerular number/glomerular volume relationship but still indicates that prenatal kidney development attempts to achieve a filtration capacity needed for a predetermined adult body size. In the figures, r denotes Pearson linear regression

The median birth weight for all subjects was 3.25 kg (IQR, 2.87–3.63) with no significant difference by race (*P* = 0.66) but with females having lower birth weight than males (*P* = 0.04). For subjects 18 years or older, there was a significant direct correlation between birth weight and N_glom_ (r = 0.292, *P* < 0.001; Spearman nonparametric correlation: r_s_ = 0.257, *P* < 0.001) with each 1 kg increase in birth weight predicting an increase of 132,446 glomeruli (95% confidence interval [CI], 71,825–193,066) (Fig. 3A). There were also direct correlations between height and birth weight (r = 0.218, *P* < 0.001; r_s_ = 0.259, r < 0.001) with each kilogram increase in birth weight predicting an increase of 4.8 cm in height (95% CI, 2.2–7.4 cm) and between height and N_glom_ (r = 0.131, *P* = 0.03; r_s_ = 0.149, *P* = 0.01) with each 1.0 cm increase in height predicting an increase in 3,737 glomeruli (95% CI, 398–7,078) (Fig. 3B, C).

V_glom_ increased with age and reached adult size at about 20 years of age. The median adult V_glom_ was 7.1 μm^3^ × 10^6^ (IQR 5.5–8.9) with a more than 6‐fold range from 2.7 to 17.0 μm^3^ × 10,^6^ and with African Americans having significantly larger V_glom_ than whites (*P* < 0.001). There was an inverse correlation between N_glom_ and V_glom_ (r = −0.409, *P* < 0.001) with each decrease of 46,067 N_glom_ predicting an increase of 1.0 μm^3^ × 10^6^ in V_glom_ (95% CI, −57,895 to −34,232) (Fig. 3D). The product of N_glom_ and V_glom_ represents V_glom_total a measure of the filtration capacity of the kidney that significantly increases with height (r = 0.213, *P* = 0.001) with every 1.0 cm increase in height predicting an increase of 0.89 cm^3^ of V_glom_total (95% CI, 0.40–1.37 cm^3^; Fig. 3E).

African Americans and whites had similar degrees of glomerulosclerosis and cortical fibrosis, but with the similar degree of severity occurring at a younger age among African Americans. Large vessel arteriosclerosis was similar in both races, but African Americans had significantly greater small arterial disease, a finding together with glomerular loss, which was previously shown to be associated with APOL1 risk alleles (Hoy et al., 2015; Hughson et al., 2017).

### 3.2 Clinical Associations between Disector/Fractionator Glomerular Number and Volume Determinations

Glomerulosclerosis, cortical fibrosis, and arteriosclerosis are features of nephrosclerosis that are closely linked with each other and with increasing age and hypertension (Table 2). Nephrosclerosis and hypertension predict glomerular loss, but the loss did not reach significance within the age range of our subjects. It should be noted, however, that the kidneys in the Monash series were selected on the basis of not having any severe degree of renal scarring. This is likely to have limited our ability to detect glomerular loss that was found in the aging population of living kidney donors in the Mayo Clinic study (Denic et al., 2017b).

**Table 2:** Age ≥18 years

	Age	N_glom_	V_glom_	GS	Cortfib	Arterioscl
N_glom_	−0.097					
	0.11					
V_glom_	**0.126**	**−0.384**				
	**0.04**	**<0.001**				
GS	**0.606**	**−0.193**	**0.126**			
	**<0.001**	**0.001**	**0.04**			
Cortfib	**0.469**	**−0.149**	**0.191**	**0.676**		
	**<0.001**	**0.01**	**<0.01**	**<0.001**		
Arterioscl	**0.533**	−0.081	**0.204**	**0.554**	**0.557**	
	**<0.001**	0.18	**0.001**	**<0.001**	**<0.001**	
HTN	**0.379**	**−0.128**	**0.346**	**0.405**	**0.420**	**0.551**
	**<0.001**	**0.03**	**<0.001**	**<0.001**	**<0.001**	**<0.001**

Spearman pairwise correlation between clinical and structural features of kidneys in the Monash series. With the cells, the upper numbers are Spearman regression coefficients and the lower numbers are *P* values.

N_glom_, number of glomeruli in right kidney; V_glom_, mean glomerular volume (μm^3^ × 10^6^); GS, glomerulosclerosis; Cortfib, cortical fibrosis; Arterioscl, arteriosclerosis, intimal thickening in interlobular renal arteries. Htn, hypertension, positive or negative.

Birth weight had no association with hypertension in either race, and Table 3 shows that there was no significant difference in N_glom_ between hypertensive and non‐hypertensive African Americans. This absence of an association of lower N_glom_ with hypertension among African Americans is further emphasized by the identification of 11 African Americans who developed hypertension before 30 years of age (Hughson et al., 2014). The median N_glom_ for these 11 subjects was 993,299 (IQR 750,602–1,138,453) and was not significantly different than the median N_glom_ of either non‐hypertensive (*P* = 0.75) or hypertensive African Americans (*P* = 0.65).

**Table 3:** Hypertension status ≥18 years old

BP status	N_glom_	*P*	Birth weight	*P*
	African American			
Non‐hypertensive	879,095 (768,894–1,154,116)	0.32	3.25 (2.79–3.75)	0.46
Hypertensive	893,357 (684,513–1,044,946)		3.32 (2.91–3.75)	
	White			
Non‐hypertensive	978,463 (737,957–1,157,012)	0.04	3.25 (2.89–3.48)	0.61
Hypertensive	833,102 (662,042–1,082,616)		3.37 (2.80–3.50)	

Comparisons of glomerular number (N_glom_) and birth weight by race. Values expressed as median and (IQR).

White hypertensives had significantly fewer glomeruli than non‐hypertensives (Table 3). In logistic regression, white hypertension was significantly related to age (odds ratio 1.06, *P* < 0.001) and female sex (odds ratio 0.32, *P* = 0.01) but not BMI (odds ratio 1.02, *P* = 0.20) or N_glom_ (odds ratio 0.99, *P* = 0.06). For sex alone, lower N_glom_ was significant for 13 hypertensive females (*P* = 0.02) but not for 35 hypertensive males (*P* = 0.15). When age was examined as a single variable, hypertensive white women were significantly older than those without hypertension (hypertensive, 51.5 ± 13.2 years; non‐hypertensive, 41.0 ± 18.4 years; *P* = 0.03). The findings suggest that the lower N_glom_ in hypertensive white women was likely to be an age‐related nephron loss and probably not a primary factor in the elevated blood pressure.

There are several factors that contribute to glomerular volume including race and hypertension (Table 4). In multiple linear regression of subjects 18 years or older, larger V_glom_ was determined by African American race (contributed a 16% increase in V_glom_ over whites, *P* < 0.01), male sex (contributed an 11% increase in V_glom_ over females, *P* = 0.045), higher BMI (obesity contributed a 26% increase in V_glom_ over normal or low body weight, <0.001), hypertension (contributed a 20% increase in V_glom_ over normotension, *P* < 0.001), and lower N_glom_ (contributed a 27% increase in V_glom_ over average or high N_glom_, *P* < 0.001). African American race, hypertension, obesity, and aging are significantly related to chronic kidney disease (Whaley‐Connell et al., 2008), and the stereological data indicate that glomerular volume may play a role in that association possibly as a reflection of an absolute deficit of nephrons or a deficit that is relative to an individual's metabolic demands (Elsherbiny et al., 2014; Hughson et al., 2014; Denic et al., 2017b).

**Table 4:** Average glomerular volume (V_glom_) and total glomerular volume (V_glom_total) ≥18 years old

V_glom_ (μm^3^ × 10^6^)	African American	*P*‐value	White	*P*‐value	*P*‐value race
Non‐hypertensive	6.60 (5.46–8.27)	<0.001	5.99 (4.62–7.71)	<0.0001	0.06
Hypertensive	8.31 (6.84–10.16)		7.45 (6.21–9.06)		0.06
V_glom_total (cm^3^)					
Non‐hypertensive	6.2 (5.0–7.7)	0.01	5.6 (4.2–6.9)	0.15	0.06
Hypertensive	7.2 (5.4–9.7)		6.4 (5.2–7.1)		0.008

Comparisons by race and hypertension status. Values expressed as median and (IQR).

### 3.3 Comparison of the Disector/ Fractionator and Renal CT Angiogram and Pretransplantation Biopsy(CT/Biopsy) Results

The Mayo Clinic group used the CT/biopsy method to assess 1,388 kidney donors ranging from 18 to 75 years old (Elsherbiny et al., 2014; Denic et al., 2017a, 2017b). Several publications resulted in which subjects had pre‐transplant metabolic studies including iothalamate determined GFRs.

There is a remarkable similarity in estimates of single kidney glomerular number between the Monash series (disector/fractionator stereology) and the Mayo Clinic study (CT/biopsy method). These estimates are compared by age range using non‐paired *t*‐test in Table 5. Only the 40–49 year and the ≥70 year old age ranges are found to be significantly different. The Monash series data did not show as steep a decline in N_glom_ after the age 40 years nor such a marked loss of glomeruli after 70 years of age as that from the Mayo Clinic, but the Monash series had very few kidneys from persons 65 years of age or older.

**Table 5:** Single kidney glomerular number by age

Age, years	n	Monash series	n	Mayo Clinic study	*P* a
18–29	45	1,000,292 ± 299,223	190	970,000 ± 430,000	0.66
30–39	62	937,782 ± 332,707	339	930,000 ± 350,000	0.86
40–49	88	950,225 ± 303,721	417	850,000 ± 360,000	0.02
50–59	58	901,382 ± 303,566	300	810,000 ± 360,000	0.07
60–64	10	901,350 ± 283,732	73	750,000 ± 310,000	0.15
65–69	13	778,118 ± 145,619	56	720,000 ± 260,000	0.44
≥70	7	836,486 ± 461,751	13	480,000 ± 170,000	0.02
	283		1,388		

Values expressed as median ± SD.

a Unpaired *t*‐test. Significance assumes normal distribution of data.

These are minor age differences that do not detract from important similarities that include a direct correlation between height and glomerular number, an inverse correlation between glomerular volume and glomerular number, and an increased glomerular volume with increased BMI. The Monash series found that birth weight was related to both height and glomerular number suggesting that birth weight was essentially “right sizing” the number of glomeruli in a kidney for an individual's adult height. The Mayo Clinic study showed an absence of a relationship between glomerular number and mild hypertension, while the Monash series findings showed no significant association between glomerular number and hypertension among African Americans of either sex nor among white males.

The substantial loss of glomeruli with aging that was found in the Mayo Clinic donor kidneys helps explain the large population of older persons, classified as having “ vascular disease”, but without any elevation of blood pressure or with only mild hypertension that are second only to diabetes as a cause for entry into US ESRD programs (Klag et al., 1996; US Renal Data System, 2013). The Mayo Clinic investigations also found that donors with a family history of ESRD had fewer and larger glomeruli than those without any family history, a link that contributes to the studies of Lackland et al. (2000) that identified low birth weight, and by inference low glomerular number, as a risk factor for ESRD (Elsherbiny et al., 2014; Denic et al., 2017b).

By iothalamate clearances, the Mayo Clinic investigators were able to estimate SNGFRs. In persons younger than 70 years, SNGFR was not significantly different across the range of glomerular number, but it did increase with larger BMI and with tall height (≥190 cm). Persons 70–75 years old had a substantial loss of glomeruli and SNGFR increased presumably as an adaptation to glomerular loss (Denic et al., 2017b).

In kidneys with low N_glom_, the reciprocity between lower glomerular number and larger volume enhances glomerular mass and supports increased glomerular filtration. Additionally, the larger glomerular volume attenuates the perfusion pressure of individual glomeruli and the potential for glomerular hypertension. This has been studied in transplant donors, where despite increased SNGFR in the remaining kidney, glomerular hypertrophy compensated for the reduction in glomerular mass, and estimates of glomerular capillary pressure remained constant (Blantz and Steiner, 2015; Lenihan et al., 2015).

The convergence of BMI and glomerular number may be a factor in the development of obesity‐related glomerulopathy. With low glomerular number, a threshold may be reached in the capacity of glomeruli to adaptively enlarge with larger BMI, and increased glomerular perfusion may eventually result in glomerular hypertension. A similar phenomenon may occur with aging, where the enlargement of remaining, non‐sclerotic glomeruli may be limited and eventually reach a point recognized as decompensated hypertensive injury (Hughson et al., 2014).

## 4 DISCUSSION

These two studies of the US subjects showed that there was a wide range in the number of nephrons in normal kidneys and that lower glomerular number was associated with an increased mean glomerular volume. The studies also showed a decline in glomerular number with age and nephrosclerosis that is quite marked in the Mayo Clinic subjects after 70 years of age. Among Mayo Clinic subjects, an increased SNGFR was observed with acquired glomerular loss in the over 70‐year‐old group, but among younger subjects what appeared to be intrinsic differences in glomerular number and volume were not associated with alterations in SNGFR. Exceptions were seen in subjects with obesity and height ≥190 cm where SNGFR did increase in association with larger glomerular volume (Denic et al., 2017b).

These findings have important implications for understanding the risk of ESRD among the large population of kidney patients that are neither diabetic nor have any defined type of kidney disease. As many as 55% of persons entering the US ESRD programs fall into this category and are diagnosed as mildly hypertensive or have never been recognized as being hypertensive at all (Klag et al., 1996; US Renal Data System, 2013). The Mayo Clinic findings of low glomerular number and increased glomerular volume in subjects with a family history of renal disease imply that the condition may eventually be found to be a low nephron‐nephropathy (Elsherbiny et al., 2014; Denic et al., 2017b).

Obesity‐related renal disease consists of diabetic changes, glomerulomegaly with proteinuria, and focal segmental glomerulosclerosis (Kambham et al., 2001). The United States and much of the Western world has an epidemic of obesity, but obesity‐related glomerulopathy affects only a small and poorly defined proportion of the obese population. The Mayo Clinic approach to estimating glomerular number should offer a tool that could determine whether or not the nephropathy is related to nephron number.

The kidney is composed of a highly redundant system of nephrons whose numbers seem to be predetermined *in utero* to meet the metabolic demands of an also predetermined adult body size, albeit without the burden of obesity. Both birth weight and adult height are predictive of glomerular number, but because of the wide variance in glomerular number throughout the range of body height or birth weight, this requires robust numbers of subjects to demonstrate. The Monash series and Mayo Clinic studies provide those numbers and are showing that glomerular number is unlikely to be related to the initiation of hypertension, at least in young‐ and middle‐aged adults. It is doubtful that this will be regarded as a completely settled question, but interest is shifting toward the relationship between low glomerular number with its attendant large glomerular size on the risk for progressive nephrosclerosis.

The Mayo Clinic studies show that large numbers of glomeruli disappear from the aging kidney and that kidney donors having family members with ESRD have reduced numbers of glomeruli (Elsherbiny et al., 2014; Denic et al., 2017a, 2017b). A family history of ESRD is a demonstrated risk for chronic kidney disease in other family members (Freedman et al., 1997; McClellan et al., 2009). The effects of shifts in perfusion onto declining numbers of large glomeruli, and whether aging, arteriosclerotic kidneys with intrinsically low glomerular number are associated with a more rapid glomerular loss will be important queries for future research.
